# Comparison of Infant Gut and Skin Microbiota, Resistome and Virulome Between Neonatal Intensive Care Unit (NICU) Environments

**DOI:** 10.3389/fmicb.2018.01361

**Published:** 2018-06-25

**Authors:** Suchitra K. Hourigan, Poorani Subramanian, Nur A. Hasan, Allison Ta, Elisabeth Klein, Nassim Chettout, Kathi Huddleston, Varsha Deopujari, Shira Levy, Rajiv Baveja, Nicole C. Clemency, Robin L. Baker, John E. Niederhuber, Rita R. Colwell

**Affiliations:** ^1^Department of Pediatrics, Inova Children’s Hospital, Falls Church, VA, United States; ^2^Inova Translational Medicine Institute, Falls Church, VA, United States; ^3^Department of Pediatric Gastroenterology, Pediatric Specialists of Virginia, Fairfax, VA, United States; ^4^CosmosID, Rockville, MD, United States; ^5^Fairfax Neonatal Associates PC, Falls Church, VA, United States

**Keywords:** microbiome, microbiota, neonatal, NICU, environment, resistome, virulome

## Abstract

**Background:** There is a growing move to provide care for premature infants in a single family, private room neonatal intensive care unit (NICU) in place of the traditional shared space, open bay NICU. The resultant effect on the developing neonatal microbiota is unknown.

**Study Design:** Stool and groin skin swabs were collected from infants in a shared-space NICU (old NICU) and a single-family room NICU (new NICU) on the same hospital campus. Metagenomic sequencing was performed and data analyzed by CosmosID bioinformatics software package.

**Results:** There were no significant differences between the cohorts in gestational age, length of stay, and delivery mode; infants in the old NICU received significantly more antibiotics (*p* = 0.03). Differentially abundant antimicrobial resistance genes and virulence associated genes were found between the cohorts in stool and skin, with more differentially abundant antimicrobial resistance genes in the new NICU. The entire bacterial microbiota analyzed to the genus level significantly differed between cohorts in skin (*p* = 0.0001) but not in stool samples. There was no difference in alpha diversity between the two cohorts. DNA viruses and fungi were detected but did not differ between cohorts.

**Conclusion:** Differences were seen in the resistome and virulome between the two cohorts with more differentially abundant antimicrobial resistance genes in the new NICU. This highlights the influence that different NICU environments can have on the neonatal microbiota. Whether the differences were due to the new NICU being a single-family NICU or located in a newly constructed building warrants exploration. Long term health outcomes from the differences observed must be followed longitudinally.

## Introduction

The human intestinal microbiota undergoes rapid dynamic changes in the first few months to years of life and these changes are hypothesized to shape future health (Yatsunenko et al., 2012). Dysbiosis of the intestinal microbiota has been associated with a wide range of diseases in humans, from gastrointestinal illnesses (Frank et al., 2007; Manichanh et al., 2012; Collins, 2014) to metabolic (Tilg and Kaser, 2011), atopic (Fujimura et al., 2014; West et al., 2015), and even neurodevelopmental conditions (Hsiao et al., 2013). Infants born prematurely and in the neonatal intensive care unit (NICU) are particularly vulnerable and have been shown to exhibit aberrant microbiota development, including harboring high levels of antimicrobial resistance genes (Costello et al., 2013; La Rosa et al., 2014; Gibson et al., 2016; Gasparrini et al., 2016). Moreover premature infants are at risk of developing conditions associated with microbiota dysbiosis, both in the NICU, such as life threatening necrotizing enterocolitis (Pammi et al., 2017a), and later in life (Boyle et al., 2012). In addition, it is also recognized that the human skin microbiome undergoes critical maturation in infancy (Capone et al., 2011) and can be influenced by prematurity (Pammi et al., 2017b). Aberrant development of the skin microbiome has the potential to allow pathogen colonization and has also been associated with the development of atopic dermatitis (Kennedy et al., 2017).

It is known that the environment influences the human microbiota with many studies showing geographical differences in child microbiota development (Fallani et al., 2010; Echarri et al., 2011; Yatsunenko et al., 2012). It has been demonstrated that microbes of the NICU resemble those found in the gut of premature infants showing the impact that this unique environment can have on microbiota development (Brooks et al., 2014). Moreover bacterial diversity from the environment can differ between different NICUs even located within a close geographical distance (Hewitt et al., 2013).

There is a growing demand to care for premature infants in a single-family, private room NICU rather than a traditional shared-space, open bay NICU, with some evidence showing decreased nosocomial infections, shorter length of stay, and lower cost of care, especially when nosocomial infections were considered (Ortenstrand et al., 2010; Domanico et al., 2011; Stevens et al., 2014; Sadatsafavi et al., 2017). It is known that the NICU environment can significantly influence the gut microbiota of premature infants. However, the effect of a shared-space NICU versus a single-family room NICU on the infant microbiota and incidence of antimicrobial resistance genes is unknown. The aim of this pilot study was to explore the composition, diversity and differences, if any, in the gut and skin microbiome, community resistome, and virulome among infants dwelling in a single-family room NICU versus a shared-space NICU.

## Materials and Methods

### Study Participants

Subjects were drawn from a larger cohort in an ongoing study entitled “The neonatal intestinal microbiome: impact on infant and early childhood health and disease” within the Inova Health System, Inova Fairfax Hospital, Falls Church, Virginia (IRB approval #15-1945). Neonates with an anticipated length of stay within the NICU of over 5 days were recruited within 3 days of life. Detailed maternal, pregnancy and delivery data were collected. While in the NICU infants had stool collected twice a week and frozen at -80°C within 12 h. Skin swabs were collected every 2 weeks using an E-Swab (Coplan, Murrieta, CA, United States) moistened with sterile saline gently rolled about 10 times in the groin area in the inguinal region. Swabs were frozen at -80°C within 12 h of collection. Detailed data regarding feeding, medications and health status were collected while the infants were in the NICU and when they were discharged, a health and disease survey and stool sample was collected every 3–6 months. The overall aim of the study was to identify microbial disruption indices prior to disease development.

During the course of this study the level IV Fairfax Neonatal Associates NICU at the Inova Fairfax Hospital moved from a shared-space open bay NICU (old NICU) into a single-family private room NICU in a newly constructed building that had not been previously occupied (new NICU). Subjects were selected from the cohort who had their entire NICU stay exclusively in the old NICU or the new NICU (those who spent time in both environments were excluded), had stool and skin samples available and were without any major congenital anomalies.

### Samples and DNA Extraction

Stool samples collected from subjects at 2 weeks of life and at the time of their discharge from the NICU were selected; the two time points were chosen to investigate whether microbiota differences between the two NICU environments developed over time or were sustained over time. Groin skin swabs taken from these subjects at 2 weeks of age were also included in the analysis. The 2 week time point was chosen as the earliest time point for both the stool and skin swab because by this time, if an infant in the study had antibiotics they had already received them and all infants in the study had started to receive some enteral feeds.

Environmental swabs were collected from both NICUs in areas where the study subjects most often spent their hospitalization and in high use areas. Swabs were collected in a similar manner as the skin swabs. Each swab was wet with sterile saline and rolled over the sample site ten times. Two control samples of methicillin-resistant *Staphylococcus aureus* and *Escherichia coli* were also included in the analysis.

Each sample underwent the following preparation prior to extraction. Samples stored at -80°C were removed from storage 1 to 2 h before DNA extraction and thawed on ice. Each sample was suspended in ASL buffer (Qiagen, Valencia, CA, United States) if stool or ATL buffer if a swab (Wang et al., 2008; Khelaifia et al., 2013; Fan et al., 2014). Samples were transferred into mechanical lysis matrix tubes (MP Biomedical, Santa Ana, CA, United States). All samples were homogenized for 10 min on an oscillating vortexer (Mo Bio, Carlsbad, CA, United States) at maximum speed and placed briefly in a flash spinner to remove excess liquid from the cap (Wang et al., 2008; de Boer et al., 2010; Salonen et al., 2010; Khelaifia et al., 2013; Fan et al., 2014). Twenty five microliters of lysozyme was added at 20 mg/mL to the sample tubes and inverted ten times to mix (Wang et al., 2008). Samples were placed on a rotating heat block at 95°C for 5 min at 2000 rpm and cooled on ice for 2–5 min. Samples were centrifuged at 20,000 ×*g* for 2 min and the supernatant was removed and placed in a new 2 mL Eppendorf tube without disturbing the pellet and matrix lysis tube beads (Wang et al., 2008; Khelaifia et al., 2013; Moles et al., 2013). Then one Inhibit X tablet (Qiagen, Valencia, CA, United States) was added to each tube and vortexed until the tablet completely dissolved and incubated at room temperature for 3 min (Wang et al., 2008). Samples were centrifuged at 20,000 ×*g* for 2 min; supernatant was removed and put into a clean 1.5 mL Eppendorf tube, and centrifuged again at 20,000 ×*g* for 3 min (Wang et al., 2008). To finish extraction on the EZ1 (Qiagen, Valencia CA, United States), 400 uL were placed into an extraction tube and loaded onto the machine using the EZ1 DSP kit with the viral extraction protocol card. Each sample was cleaned and concentrated using the NucleoSpin gDNA Clean-up XS kit (MACHEREY-NAGEL GmbH & Co. KG).

### Sequencing and Metagenomic Analyses

DNA samples were normalized in 50 μL of nuclease-free water using 0.0–0.29 ug of input materials on the Biomek FX liquid handler. For each sample, an input of 0.5 ng was used in the tagmentation reaction, followed by 13 cycles of PCR amplification using Nextera i7 and i5 index primers and 2× Kapa master mix per the modified Nextera XT protocol. The PCR products were purified using 1.0× speed beads and eluted in 15 ul of nuclease-free water. The final libraries were then quantitated using the picogreen fluorometric assay (100× final dilution) and the concentrations were in the range of 0.1–4.0 ng/ul. The libraries were pooled based on their concentrations as determined by picogreen and loaded onto a high sensitivity chip run on the Caliper LabChipGX; the base pair size reported was in the range of 301–680 bp. Libraries were sequenced using Illumina HiSeq v3 chemistry for 100 bp single end reads with the aim to generate 40 M reads per sample.

Unassembled metagenomic sequencing reads were directly analyzed using the CosmosID bioinformatics software package (CosmosID Inc., Rockville, MD, United States) as described previously (Hasan et al., 2014; Lax et al., 2014; Ponnusamy et al., 2016) to achieve microbial identification to the species, subspecies, and/or strain level and quantification of organism’s relative abundance. CosmosID is a microbial genomics platform focused on rapid characterization of microorganisms, pathogens and anti-microbial resistance for infectious disease identification, food safety inspections, pharmaceutical discovery, public health surveillance and microbiome analysis. CosmosID bioinformatics utilizes high performance data mining algorithms and highly curated dynamic comparator databases (GenBook^®^) that are readily accessible by cloud interface. The curated databases provide extremely fine resolution in identification, discrimination of pathogens from ‘near neighbors’, and accurate measurement of relative abundances. CosmosID utilizes a high performance data-mining K-mer based algorithm that rapidly disambiguates hundreds of millions of short reads of a metagenomic sample into the discrete microorganisms engendering the particular sequences. While the tools are flexible and can be used to compare whole genomes, the principal software pipeline has been optimized for processing unmapped and unaligned raw sequence reads of lengths less than 100 basepairs. The pipeline has two separable comparators. The first consists of a pre-computation phase and a per-sample computation. The input to the pre-computation phase is a reference microbial database, and its output is a whole genome phylogeny tree, together with sets of fixed length k-mer fingerprints (biomarkers) that are uniquely identified with distinct nodes of the tree. The second per-sample, computational phase searches the hundreds of millions of short sequence reads against the fingerprint sets in minutes. The resulting statistics are analyzed to give fine-grain composition and relative abundance estimates at all nodes of the tree. The second comparator uses edit distance-scoring techniques to compare a target sample with a reference set. The algorithm provides similar functionality to BLAST but sacrifices some recall precision for a one or two order of magnitude processing gain. Overall classification precision is maintained through aggregation statistics. This second comparator may be used independently of the first. However, enhanced discriminatory power is achieved by running the comparators in sequence. The first comparator finds reads in which there is an exact match with an n-mer uniquely identified with a set of reference strains; the second comparator then statistically scores the entire read against the reference to verify that the read is indeed uniquely identified with that set. Similarly, the community resistome and virulome, the collection of antibiotic resistance and virulence genes respectively in the microbiome, were also profiled by querying unassembled sequence reads against CosmosID curated antibiotic resistance and virulence gene databases.

### Comparative Statistical Analysis

Demographic and clinical features were compared between the two NICU cohorts using Chi-squared or Fischer’s exact test, as appropriate, for categorical variables and an unpaired two tailed *T*-test for continuous variables. Analyses of the sequencing data included generation of heatmaps based on the relative abundance of each microorganism (%) in each sample using the NMF R software package (Gaujoux and Seoighe, 2010). Likelihood ratio testing was performed using a parameterization of the Dirichlet-Multinomial distribution developed for comparisons of whole genome shotgun metagenomic datasets (La Rosa et al., 2012) using the data subsampled to 10 million reads to avoid any statistical bias due to different sample sizes. Similarity index calculations were performed as described (Tarkkanen et al., 2009) using the Pearson correlation and boxplots were computed using the ggplot2 R library (McGill et al., 1978). Principal coordinate analysis (PCoA) was performed using the Bray-Curtis distance measure and clustered using the Partitioning Around Medoids (PAM) algorithm (Kaufman and Rousseeuw, 1987). Resistome analysis was performed by identification of antibiotic-resistance genes based on percentage of gene coverage for each gene as a function of the gene-specific read frequency in each sample. Statistical analyses were performed using a one-tailed Student’s *T*-test.

## Results

### Demographics and Clinical Factors

A total of 32 infants were included in this study, 14 from the old NICU and 18 from the new NICU. **Table 1** shows demographic and key clinical features for the two groups. There were no significant differences between the two cohorts in gestational age, length of stay or delivery mode. There was a significantly greater amount of antibiotic use during NICU stay in the old NICU, compared with the new NICU (12/14 infants in old NICU vs. 8 /18 infants in new NICU, *p* = 0.03). Analysis of the specific classes of antibiotics used showed more 3rd generation cephalosporin use in the old NICU compared with the new NICU (4/14 infants in old NICU vs. 0/18 infants in new NICU, *p* = 0.03). No difference was detected between maternal antibiotic use in pregnancy between the two cohorts nor was there any difference between the number of infants who received total parenteral nutrition (TPN) or some maternal or donor breast milk. However, more infants in the old NICU received exclusive maternal or donor breast milk (after TPN use if needed) compared with the new NICU (11/14 infants in old NICU vs. 7/18 infants in new NICU, *p* = 0.04).

**Table 1:** Comparison of demographics and clinical factors between the Old and New NICU.

	Old NICU	New NICU	*p*-value
Number of subjects	14	18	
Mean gestational age in weeks	31.4	33.2	0.16
Average length of stay in days	33.5	33.2	0.52
**Mode of delivery**			
Vaginal delivery	5	1	0.06
Cesarean Section	19	17	
**Infant antibiotic use ever**			
Yes	12	8	**0.03**
No	2	10	
**Infant type of antibiotic use**			
Ampicillin	10	8	0.16
Gentamicin	10	8	0.16
1^st^ generation cephalosporin	1	1	1.00
2^nd^ generation cephalosporin	1	0	0.44
3^rd^ generation cephalosporin	4	0	**0.03**
Vancomycin	0	1	1.00
Piperacillin/tazobactam	0	1	1.00
**Maternal use of antibiotics during pregnancy (notincluding those given at Cesarean Section)**			
Ever (may include more than 1 course)	1	3	0.61
Ampicillin	1	0	0.44
Azithromycin	1	0	0.44
1^st^ generation cephalosporin	0	3	0.24
Nitrofurantoin	0	1	1.00
**Nutrition**			
Ever received TPN	12	12	0.41
Primarily receiving maternal or donor breast milk only (excluding fortifiers)	11	7	**0.04**
Receiving some maternal or donor breast milk	11	17	0.30

*Bolded values are statistically significant.*

### Metagenomic Sequencing

**Table 2** shows the number of each type of sample successfully sequenced from each NICU cohort and also lists the locations of the environmental swabs obtained. Area 1 and area 2 of the new NICU represent two areas separated geographically that the babies in this cohort most frequently stayed in. Between sample types, there was no significant difference in the amount of DNA extracted between cohorts.

**Table 2:** Number of each sample type and comparison of the quantity of DNA extracted from each sample type from the Old and New NICU.

	Old NICU (n)		Mean DNA extracted in ng	New NICU (n)		Mean DNA extracted in ng	*p*-value for difference in DNA between 2 cohorts
2 week stool samples	9	7.09		13		3.72	0.48
Discharge stool samples	12	16.66		14		4.90	0.33
Subjects with both stool samples	9	n/a		10		n/a	n/a
Skin samples	11	4.46		10		3.16	0.53
Environmental samples	6	1.26		10		0.49	0.08
Location of environmental samples		∙ Sink			**Area 1**	
		∙ Vitals monitor			∙ Sink	
		∙ Nurse station			∙ Vitals monitor	
		∙ Light switch			∙ Bedside cart	
		∙ Foam pump			∙ Nurses station	
		∙ Baby swing chair			∙ Foam pump	
					**Area 2**	
					∙ Sink	
					∙ Vitals monitor	
					∙ Bedside cart	
					∙ Nurses station	
					∙ Foam pump	

Metagenomic sequencing of DNA extracted for skin, stool and environment swabs generated 22–67 million reads per sample with an average of 46 million reads per sample. Sequence data were deposited in to NCBI Bioproject under accession number PRJNA417283.

### Species Alpha Diversity

**FIGURE 1:**
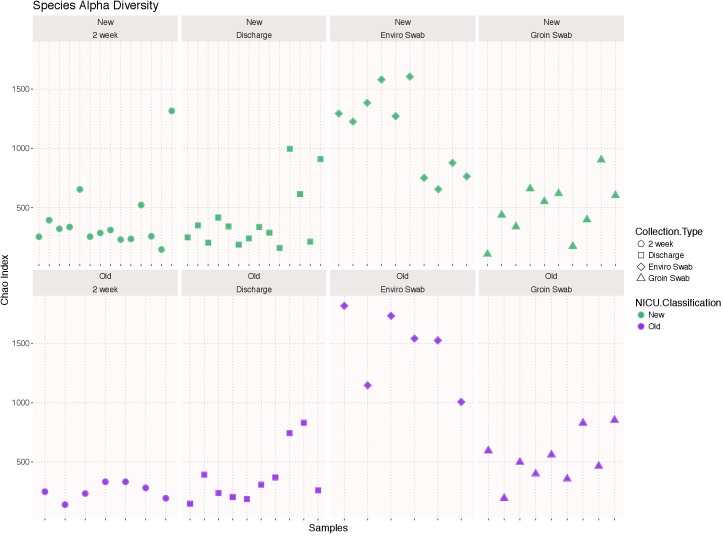
Alpha diversity using the Chao 1 index of each sample type (2 week stool, discharge stool, environmental swabs, and groin skin swabs) in the old NICU and the new NICU. There was no significant difference in any of the four samples types between the two NICU cohorts.

There was no significant difference in species alpha diversity using the Chao 1 index, in any of the four samples types between the two NICU cohorts (**Figure 1**). However, noticeable variation was observed within the sample types, i.e., environment and groin swabs demonstrated greater alpha diversity than that of 2 week and discharge stool samples.

### Comparison of the Entire Bacterial Microbiota

**FIGURE 2:**
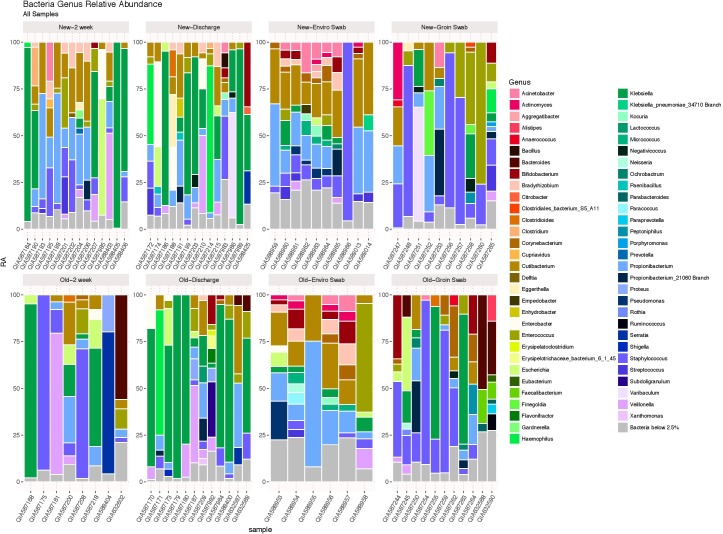
The bacterial microbiome at the taxonomic level of genus in each sample type in the old NICU and the new NICU samples. Using the likelihood ratio test, there was no difference between the old and new NICU in 2 week and discharge stool samples (*p* = 1.00), but there was a significant difference between groin skin swab (*p* = 0.0001) and environmental swab samples (*p* = 0.0003) between the two cohorts.

Using the likelihood ratio test, comparison of the entire bacterial community at the genus level showed no difference between infants from the old and new NICU in 2 week and discharge stool samples (*p* = 1.00), but did show a significant difference between skin swab (*p* = 0.0001) and environmental swab samples (*p* = 0.0003) for the two cohorts (**Figure 2**). There was no significant difference between 2 week and discharge stool samples from the old NICU but there was a significant difference between 2 week and discharge stool samples from the new NICU (*p* = 0.0006).

### Specific Taxa Comparison

Heat maps showing relative abundance of genera represented in the 2-week and discharge stool samples are provided in **Supplementary Figure S1A**, stratified by if the infant was primarily receiving breast milk at the time of the sample collection. Specific taxa of interest were noted, with no significant differences observed between *Bifidobacterium, Lactobacillus, Enterobacteriaceae, Staphylococcaceae, and Ureaplasma* in both 2 week and discharge stool samples for the two NICU cohorts or by breast milk status. Heat maps showing relative abundance of genera in skin samples and environmental samples between the two cohorts are provided in **Supplementary Figures S1B,C**, respectively. The following specific taxa of interest were analyzed for skin samples, with no significant difference between the two cohorts: *Proteobacteria, Firmicutes, Actinobacteria, Staphylococcus, Staphylococcus aureus*. The following specific taxa of interest were examined in the environmental samples and no significant difference was observed between the two cohorts: *Streptococcus, Staphylococcus, Neisseria, Enterobacteriaceae, Klebsiella, Bacteroides fragilis, Escherichia coli*.

**Supplementary Figure S2** shows a PCoA biplot of bacterial content at the taxonomic level of class for 2 week and discharge stool samples, comparing old and new NICU after removal of outliers. Interestingly outliers from the old and new NICU, shown in the bottom left of **Supplementary Figure S2** reveal clustering on different sides of the plot. Arrows point in the direction of highest correlation between taxa and sample clusters and are colored by phyla.

### Antimicrobial Resistance Genes

**FIGURE 3:**
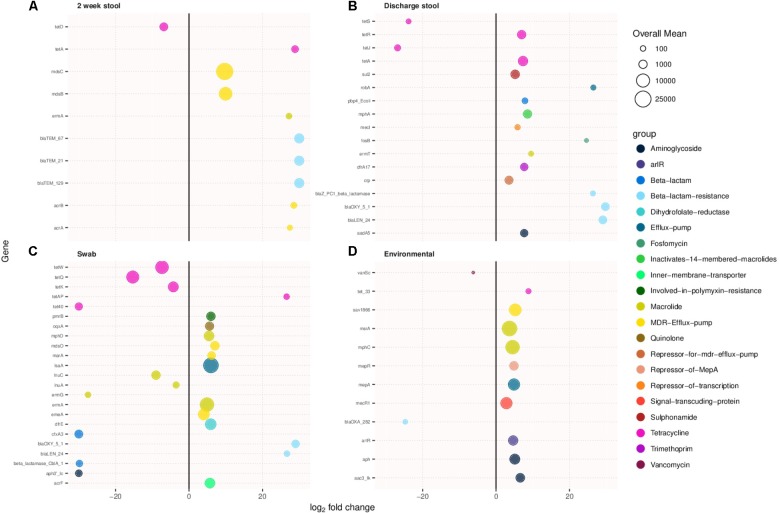
Antimicrobial resistance genes showing more than two-fold log change in prevalence in samples collected from the new NICU compared to the old NICU samples. Antibiotic resistance genes were more abundant in the new NICU, compared to the old NICU, in all sample types. **(A)** 2 week stool, **(B)** discharge stool, **(C)** skin swab, and **(D)** environmental swab.

Antimicrobial resistance genes were compared between sample types of the two cohorts. **Figures 3A–D** show antibiotic resistance genes with over a two log difference between old and new NICU for the 2 week stool, discharge stool, skin swabs, and environmental swabs, respectively. More antibiotic resistance genes were differentially abundant in samples collected from the new NICU compared to the old NICU for all sample types. Of note, in stool samples, both 2 week and discharge, and skin swabs, beta lactam resistance genes were notably more differentially abundant in the new NICU samples than the old NICU. Due to the difference in antibiotic use seen between the 2 cohorts (higher use in old NICU), differentially abundant antimicrobial resistance genes were compared between only those who received antibiotics in the old and new NICU, and between those who did not receive antibiotics in the old and new NICU (**Supplementary Figures S3A,B**, respectively). In those who received antibiotics, more differentially abundant genes were seen in the new NICU compared with the old NICU in the 2 week stool sample only. Of note, in those who received antibiotics, different differentially abundant genes were identified compared with the cohort as a whole, with tetracycline resistance genes more abundant in the new NICU. In comparing those without antibiotic exposure, differentially abundant antimicrobial resistance genes were only seen in skin swabs, with more genes in the new NICU. Due to the difference seen in infants primarily receiving breast milk (higher in the old NICU), differentially abundant antimicrobial resistance genes were compared between only those who primarily received breast milk in the old and new NICU, and between those who did not primarily receive breast milk in the old and new NICU (**Supplementary Figures S4A,B**, respectively). In those who primarily received breast milk, more differentially abundant antibiotic resistance genes were still seen in the new NICU in the 2 week stool and skin swabs samples with a notable prominence of tetracycline and beta lactam resistance genes. In those who did not primarily receive breast milk, more differentially abundant genes were seen in the new NICU in the 2 week stool and discharge stool samples, once again with a notable difference in beta lactam resistance genes. However, in skin swabs more differentially abundant genes were seen in the old NICU in those who did not primarily receive breast milk. No individual from either cohort developed clinically detectable infection caused by an antibiotic resistant organism during their NICU stay.

### Virulence Associated Genes

**FIGURE 4:**
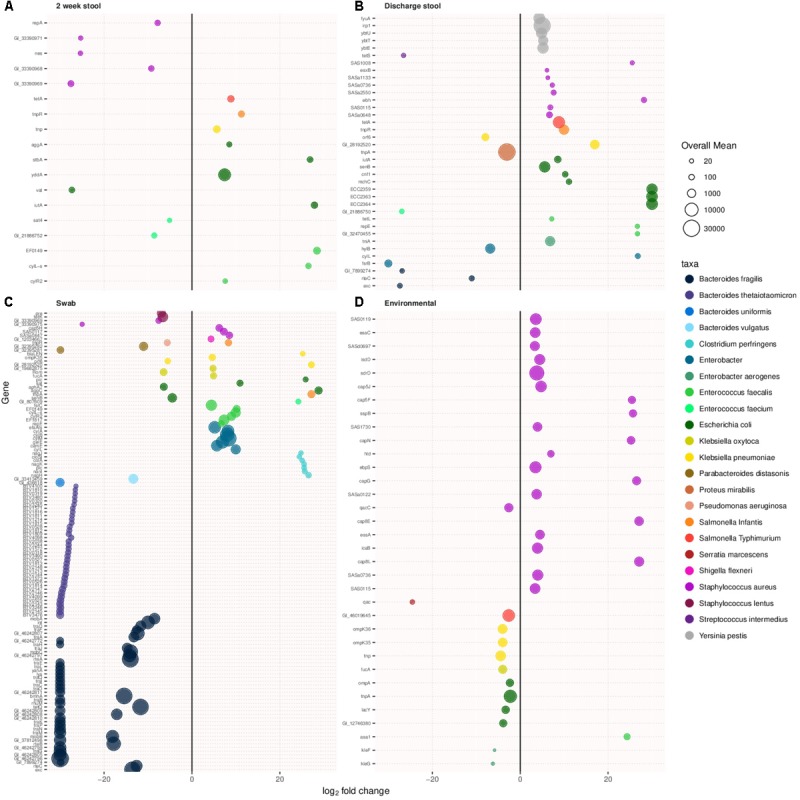
Virulence associated genes with over a log two fold change in prevalence in the new NICU compared with the old NICU samples. Differentially abundant virulence associated genes were detected in all sample types between the two NICU cohorts. **(A)** 2 week stool, **(B)** discharge stool, **(C)** skin swab, and **(D)** environmental swab.

Virulence related genes were compared between samples types between the two cohorts. **Figures 4A–D** show virulence associated genes with more than a log two difference between old and new NICU in 2 week stool, discharge stool, skin swabs, and environmental swabs, respectively. Differentially abundant virulence related genes between the two cohorts were found in each sample type. Discharge stool samples demonstrated a difference in virulence related genes typically associated with *Escherichia coli* in the new NICU and *Bacteroides fragilis* and *Enterobacter* associated virulence genes in the old NICU. In skin swabs a bigger difference was observed among *Bacteroides fragilis and Bacteroides thetaiotaomicron* associated virulence genes in the old NICU and *Enterobacter, Enterococcus faecalis* and *Clostridium perfringens* associated virulence genes in the new NICU. Environmental samples revealed a difference in *Staphylococcus aureus* related virulence factor coding genes in samples from the new NICU. However, it is important to note that as virulence genes often undergo horizontal gene transfer, it is possible that any other microbial community members and not those typically known to harbor those genes, may have been carrying these genes.

### Viruses

**FIGURE 5:**
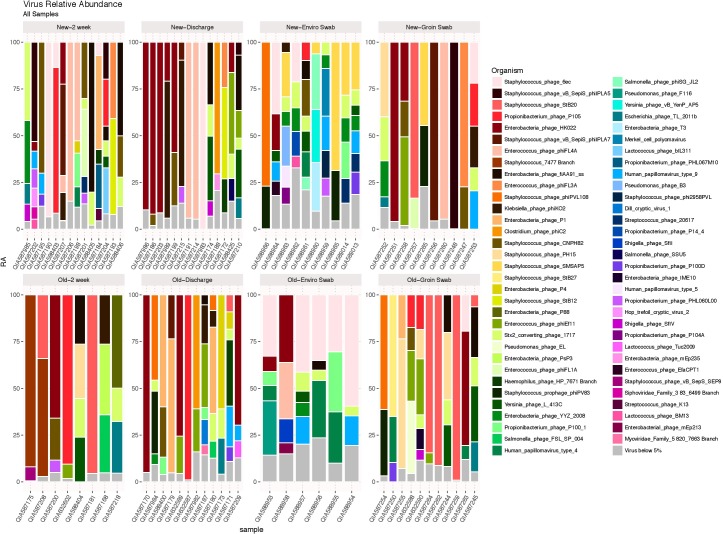
DNA viruses by sample type. DNA viruses were detected in all sample types in both cohorts and were predominantly phages.

DNA viruses were detected in all sample types in both cohorts, as shown in **Figure 5**, and consisted primarily of phages. No significant differences in 2 week stool, discharge stool and skin samples were noted between the two NICU cohorts with respect to potential human pathogenic viruses (including herpesvirus, papillomavirus, adenovirus, merckel cell virus, corona virus and respiratory syncytial virus). The environmental samples however revealed polyomaviruses were more frequently detected in samples from the old NICU compared with the new NICU (*p* = 0.017). No subject developed any clinically detectable viral infections during their stay in the NICU.

### Fungi and Parasites

**FIGURE 6:**
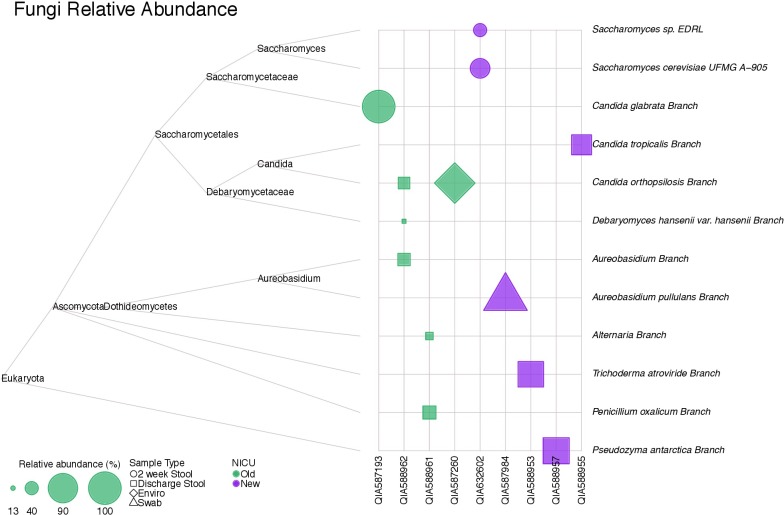
A tree map of fungi detected in samples collected in this study. Fungi were not detected in all samples, but were more common in stool samples.

**Figure 6** shows a tree map of fungi detected in samples analyzed in this study. Fungi were not present in all samples, but were more frequently detected in stool samples. A parasite, *Acanthamoeba polyphaga*, was detected in a skin swab sample from a subject from the old NICU.

### Controls

Sequencing of the controls, *Staphylococcus aureus* and *Escherichia coli*, provided reference for the analyses as a test for contamination and quality of the analyses.

## Discussion

This pilot study is the first to compare microbiome differences between infants in an open bay shared-space NICU (old NICU) and the transition to a new hospital building with a single-family room NICU environment (new NICU), both on the same hospital campus.

Notably, differentially abundant antibiotic resistance genes were observed in all sample types (stool, skin, and environment) between both cohorts, with more abundant antibiotic resistance genes detected in samples collected from the new NICU. The hypothesis was that the new single-family room NICU would have fewer antibiotic resistance genes detected in the microbiota of infants because single-family room NICUs have been shown to have decreased rates of nosocomial sepsis (Domanico et al., 2011). In addition, it is assumed that there is patient to patient transfer of microbiota, including those microorganisms with antibiotic resistance potential, via hand carriage of health workers. Open bay NICUs are hypothesized to lead to decreased hand washing frequency or less efficiency between patients whereas private rooms have readily accessible hand washing stations in each room, providing more opportunity for hand washing by healthcare workers (Saiman, 2002; Walsh et al., 2006). The results were therefore surprising, finding more abundant antibiotic resistance genes in samples collected from the new NICU, especially as there was significantly less antibiotic use in the new NICU compared with the old, with antibiotic use being a known risk factor (Gasparrini et al., 2016; Gibson et al., 2016). Specifically, cephalosporin use was less in the new NICU, even though beta lactam resistance genes were relatively more abundant in stool and skin samples in the new NICU. Even when antimicrobial resistance genes were compared in only those who had received antibiotics, differentially abundant genes were found between the two cohorts although the specific genes did differ. A possible explanation for the differentially abundant antibiotic resistance genes between samples from the two cohorts is that the resistome in this study may reflect the new NICU being located in a newly constructed building, that was previously unoccupied. A recent study by Lax et al. (2017) examined microbiota colonization and succession in a newly opened hospital, using both environmental and skin swabs. Bacterial communities on the skin of patients and from room surfaces became increasingly similar over the course of a patient’s stay and alpha and beta diversity of skin swabs was found to be only weakly associated with antibiotic usage. Moreover, Lax et al. (2017) showed in samples that underwent metagenomic sequencing, antibiotic resistance genes were more prevalent on surfaces of the room than on skin of the patient. The combined results do indicate that the microbiota of a patient is susceptible to influence of the environmental microbiota in a building. This factor may be more influential in the NICU sample comparisons of this study as newborns begin life with a very “naïve” immature microbiota and may be more vulnerable to colonization from external sources. Antimicrobial resistance genes have a survival advantage and may be more prevalent when there is less competition from lack of established bacterial communities in a new building. It is possible that over time the new NICU “matures” in terms of microbiota colonization, effecting favorable change in the community resistome. Another possible explanation of the finding is that neonates in the new NICU are potentially exposed to fewer caregivers and if a specific caregiver carries more of these genes they may be more likely to colonize the infant. However, contradicting this, it was found that antimicrobial resistance genes in the new NICU did not cluster in certain individuals and were found in all sample types across infants. Lastly, it could be hypothesized that differences in feeding and nutrition between the 2 cohorts may have contributed to the difference in antimicrobial resistance genes seen. However, more infants primarily received breast milk in the old NICU, which is thought to be a protective factor (Marks et al., 2013), yet more differentially abundant antimicrobial genes were seen in the new NICU. Indeed, even when samples from only infants who primarily received breast milk were examined overall more differentially abundant antimicrobial genes were still seen in the new NICU.

Differentially abundant virulence related genes were also found in all sample types between the two NICU cohorts. Little information has been published concerning the virulome in the NICU, except for specific microorganisms. Thus this study provides valuable new information. The specific genes detected in samples collected from the new NICU may be more reflective of a newly constructed building, without competitive microbiota or colonization, rather than a single-family NICU. It is important to note that no infant from either cohort developed any clinically detectable infections caused by antibiotic resistant organisms or organisms associated with the detected virulence factors during their NICU stay. It is imperative to repeat this study with neonates who have been in the new NICU for a longer time after opening to determine whether differences observed in our study were more reflective of a new building or of a single-family room environment. Moreover, it is key to determine whether these observed differences persist into childhood, with long term health outcomes. A recent study indicated that the high levels of antibiotic resistance in the neonatal microbiota of infants found in the NICU may normalize at 2 years of age (Moles et al., 2015). This interesting observation will be investigated for the cohorts of this study in our longitudinal investigation.

No differences were observed in alpha diversity between the two cohorts but it is notable that environmental samples in the new NICU were as diverse as those in the old NICU, given the limited time the new building was operative and able to be to colonized. Lax et al. observed that, after hospital opening, alpha diversity increased in samples collected from the surfaces of the nurses’ station, which had common human skin contact, but not for floor samples (Kaufman and Rousseeuw, 1987). In our study, overall comparison of the bacterial microbiota revealed no differences between the two cohorts, with respect to stool samples. Individual variability and small sample size may be factors, but differences were observed in both patient skin and environmental samples. It will be important to monitor ecological succession of new NICU as it matures over time and determine whether these differences persist and exert any long-term outcomes for neonates. Although no differences were detected in DNA viruses, fungi, and parasites between the two patient cohorts, this comprehensive exploration emphasizes the power of such analysis for detection of potential pathogens and characterization of the virulome and resistome. Follow up studies should employ metatranscriptomics to also address if differences in RNA viruses are detected in infants from different NICU environments.

Limitations of this pilot study include a relatively small sample size, partly due to failure in sequencing of some samples and cost of resequencing. Power calculations for the differentially abundant gene statistics were conducted using the size R package (Bi and Liu, 2016) (**Supplementary Figure S5**). Also several types of patient samples were examined (stool at 2 weeks, discharge stool samples, and skin swabs) and the discharge stool sample was collected at differing ages for each patient depending on their length of stay adding further variation to the samples. However, the findings of the study are strengthened by the fact that differences in resistome and virulome between the two cohorts were detected in all sample types. The subjects of this study were carefully clinically matched but this was limited by sample size. Nevertheless, antibiotic usage and exclusive breast or donor milk feeding did differ between the two cohorts. More recently NICU practice generally has moved away from using empiric antibiotics for premature infants, with much less use of antibiotics in the new NICU than the old NICU in our study. This makes the finding of relatively more differentially abundant antibiotic resistance genes in the new NICU more intriguing and possibly more related to being a newly constructed facility. While the vast majority of infants in the new NICU (17/18) received some breast milk or donor milk, the fact that there were more infants that received exclusive breast milk or donor milk after TPN use in the old NICU may have had some influence on the microbiomes of the two cohorts (Cong et al., 2016). Nevertheless, differences in community resistome and virulome were detected in environmental samples also and not just infant samples. While this pilot study was exploratory and descriptive by nature, with clinical factors not fully controlled in both groups and with limited sample size to enable detailed further stratification by clinical features, it is the first study of its kind to give an overview of the potential environmental influences on the microbial community and its associated resistome and viruolme. Although the aim of this study was not to build associations, this will be examined in depth as the study is expanded with more individuals and samples from both environments, and critically when the new NICU has had time to “mature” and become stable through the process of ecological succession over time. Lastly, examining longitudinal microbiome development into childhood, as possible in a longitudinal cohort study such as this, is imperative to understand whether these early changes detected have any lasting impact on childhood health.

## Conclusion

There were no differences observed in species alpha diversity between the two NICU cohorts of this study, regardless of sample type. However, differences were observed in the resistome and virulome between the two NICU cohorts, with relatively more differentially abundant antimicrobial resistance genes detected in the new NICU samples. This study highlights the influence of the NICU environment on the neonatal microbiome. Whether differences observed in the new NICU compared with the old NICU samples were related to a single-family NICU or a newly opened building warrants further exploration. Moreover, whether long term health outcomes derive from the differences in microbiomes detected between the two cohorts will be followed longitudinally in this ongoing research.

## Declarations

### Ethics Approval and Consent to Participate

This study was approved by the Inova Human Research Protection program; IRB approval #15-1945. All subjects underwent informed consent to participate.

### Availability of Data and Material

Sequence data were deposited in to NCBI Bioproject under accession number PRJNA417283.

## Author Contributions

SH, EK, KH, RB, RLB, and JN designed the study. VD, EK, and SL recruited and consented patients and collected subjects samples. AT and NaC clinically phenotyped subjects. NiC processed and quality controlled samples. PS, NH, and RC analyzed and interpreted metagenomic sequencing data. SH drafted the manuscript. All authors read and approved the final manuscript.

## Conflict of Interest Statement

The authors declare that the research was conducted in the absence of any commercial or financial relationships that could be construed as a potential conflict of interest.

## Supplementary Material

FIGURE S1(A) Heat maps showing the relative abundance of genera in 2-week and discharge stool samples between samples from the old NICU and the new NICU, stratified by if the infant was primarily receiving breast milk at the time of the sample collection.

FIGURE S1(B) Heat maps showing the relative abundance of genera in skin samples between samples from the old NICU and the new NICU.

FIGURE S1(C) Heat maps showing the relative abundance of genera in environmental samples between samples from the old NICU and the new NICU.

FIGURE S2 PCoA biplot of bacterial content at the taxonomic level of class for 2 week and discharge stool samples, comparing old and new NICU after removal of outliers. Interestingly outliers from the old and new NICU, shown in the bottom left of figure reveal clustering on different sides of the plot. Arrows point in the direction of highest correlation between taxa and sample clusters and are colored by phyla.

FIGURE S3A Antimicrobial resistance genes showing more than a two-fold log change in prevalence in samples collected from infants in the new NICU compared to the old NICU samples, in infants who had received antibiotics.

FIGURE S3B Antimicrobial resistance genes showing more than a two-fold log change in prevalence in samples collected from infants in the new NICU compared to the old NICU samples, in infants who did not receive antibiotics.

FIGURE S4A Antimicrobial resistance genes showing more than a two-fold log change in prevalence in samples collected from infants in the new NICU compared to the old NICU samples, in infants who primarily received breast milk.

FIGURE S4B Antimicrobial resistance genes showing more than a two-fold log change in prevalence in samples collected from infants in the new NICU compared to the old NICU samples, in infants who did not primarily receive breast milk.

FIGURE S5 Average power versus sample size for differential gene abundance test using false discovery rate of 0.05. π0 is the estimated proportion of genes which are not differentially abundant.
