# Supplementary figures and images for: Comparison of Infant Gut and Skin Microbiota, Resistome and Virulome Between Neonatal Intensive Care Unit (NICU) Environments
Source: Front Microbiol. 2018 Jun 25;9:1361. doi: 10.3389/fmicb.2018.01361 (PMC6026636; doi:10.3389/fmicb.2018.01361)

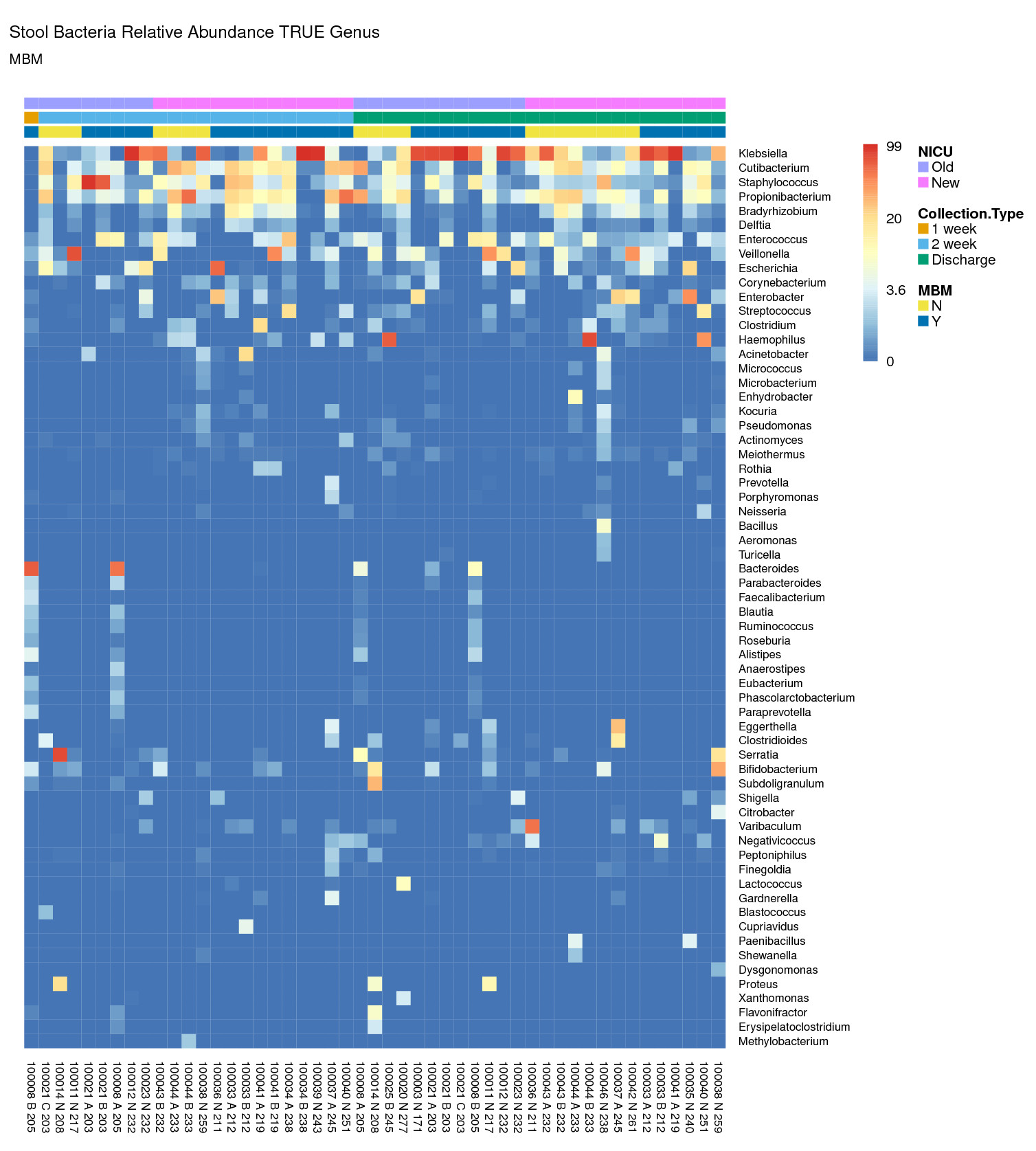

Supplement: FIGURE S1(A) — Heat maps showing the relative abundance of genera in 2-week and discharge stool samples between samples from the old NICU and the new NICU, stratified by if the infant was primarily receiving breast milk at the time of the sample collection. [file Image_1.JPEG]

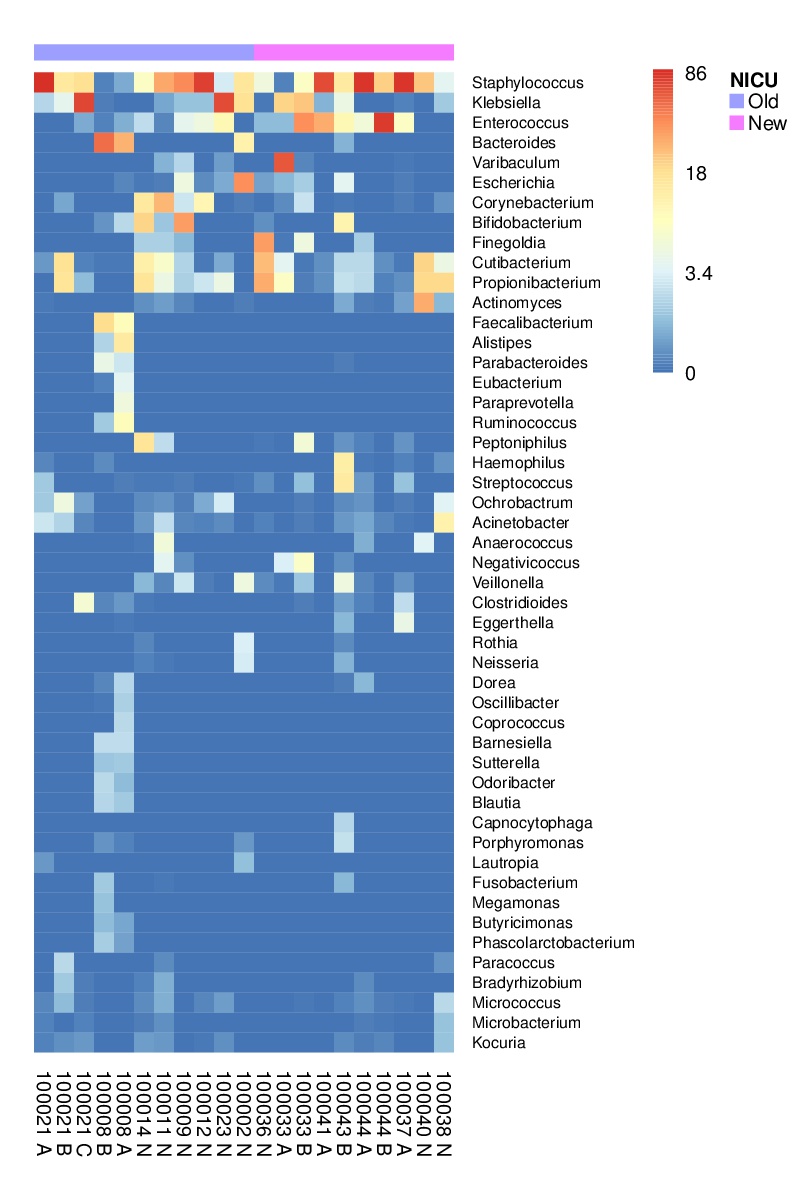

Supplement: FIGURE S1(B) — Heat maps showing the relative abundance of genera in skin samples between samples from the old NICU and the new NICU. [file Image_2.JPEG]

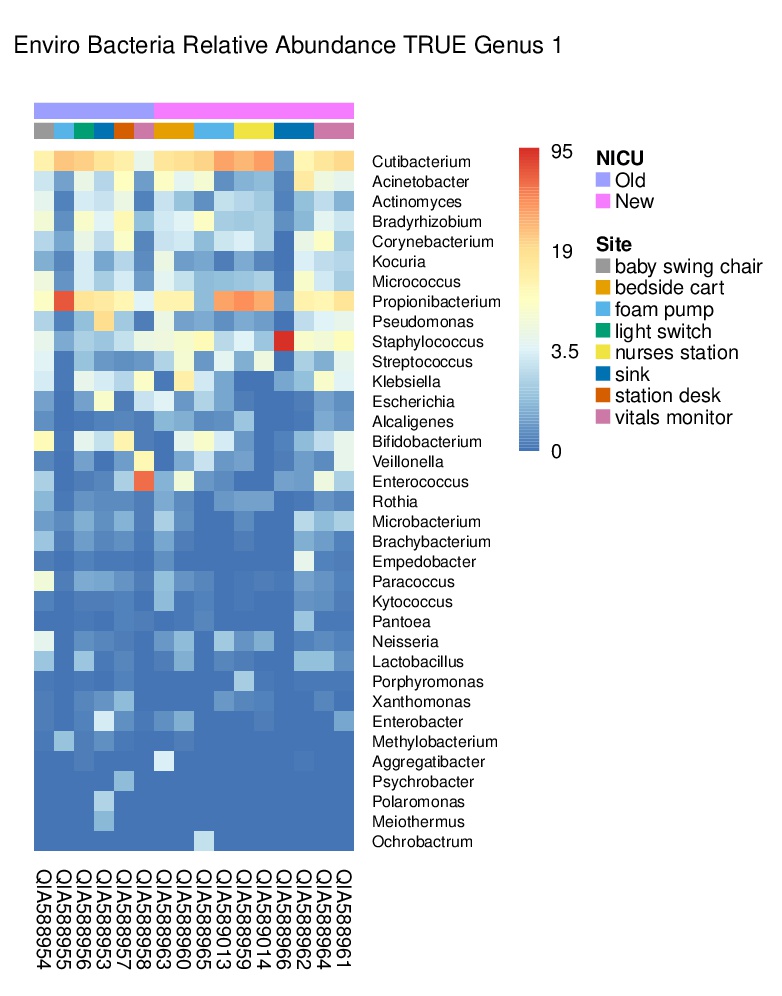

Supplement: FIGURE S1(C) — Heat maps showing the relative abundance of genera in environmental samples between samples from the old NICU and the new NICU. [file Image_3.JPEG]

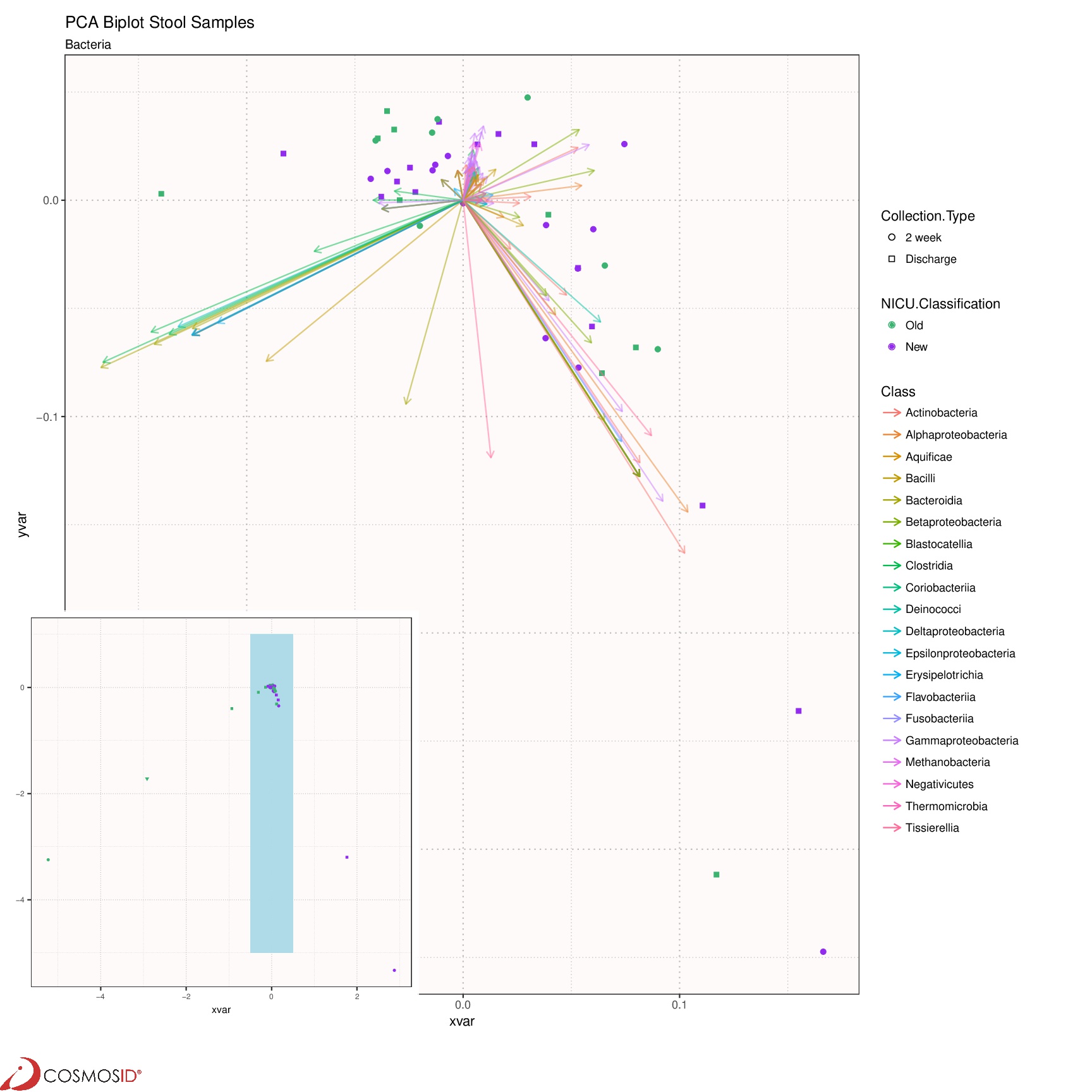

Supplement: FIGURE S2 — PCoA biplot of bacterial content at the taxonomic level of class for 2 week and discharge stool samples, comparing old and new NICU after removal of outliers. Interestingly outliers from the old and new NICU, shown in the bottom left of figure reveal clustering on different sides of the plot. Arrows point in the direction of highest correlation between taxa and sample clusters and are colored by phyla. [file Image_4.JPEG]

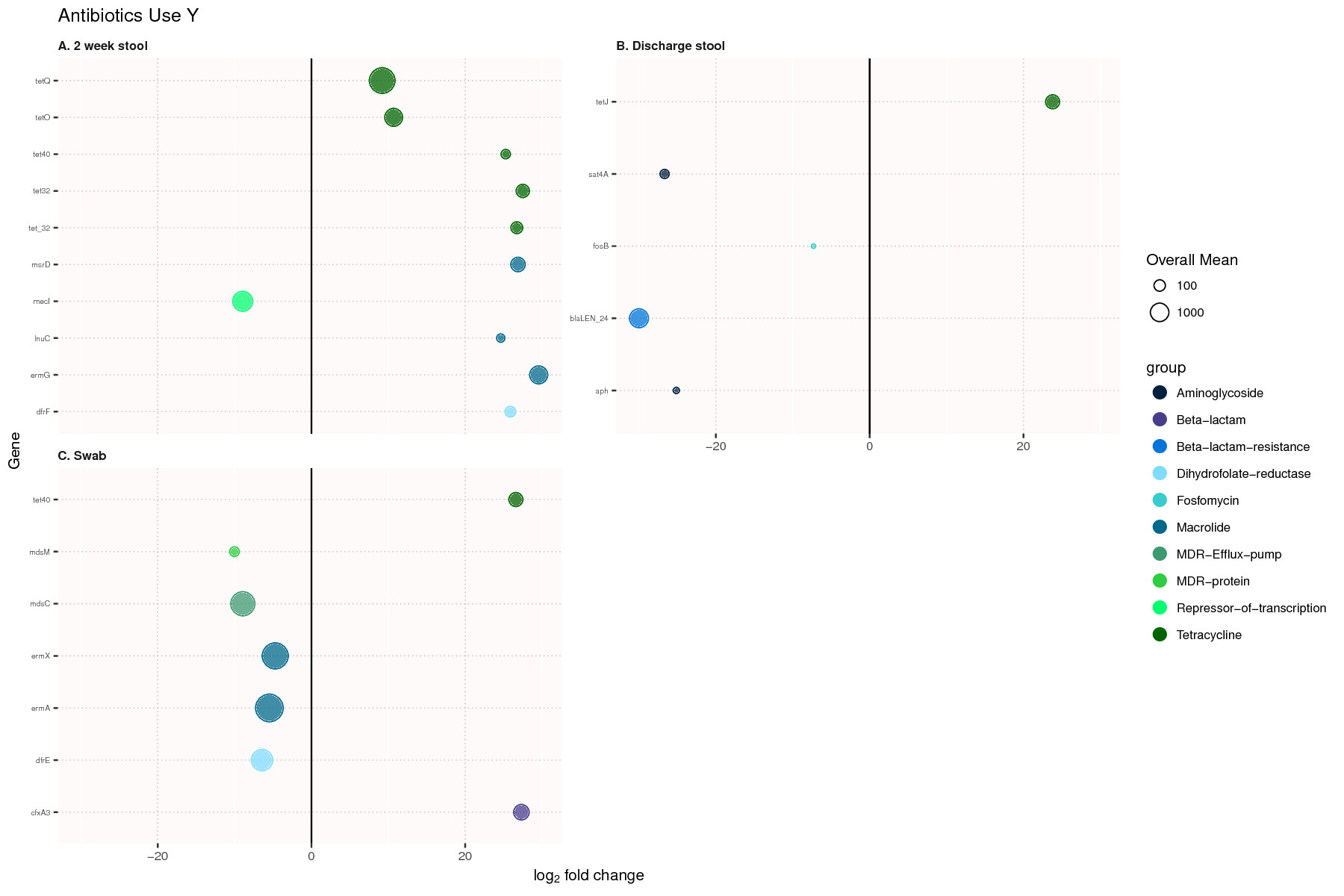

Supplement: FIGURE S3A — Antimicrobial resistance genes showing more than a two-fold log change in prevalence in samples collected from infants in the new NICU compared to the old NICU samples, in infants who had received antibiotics. [file Image_5.JPEG]

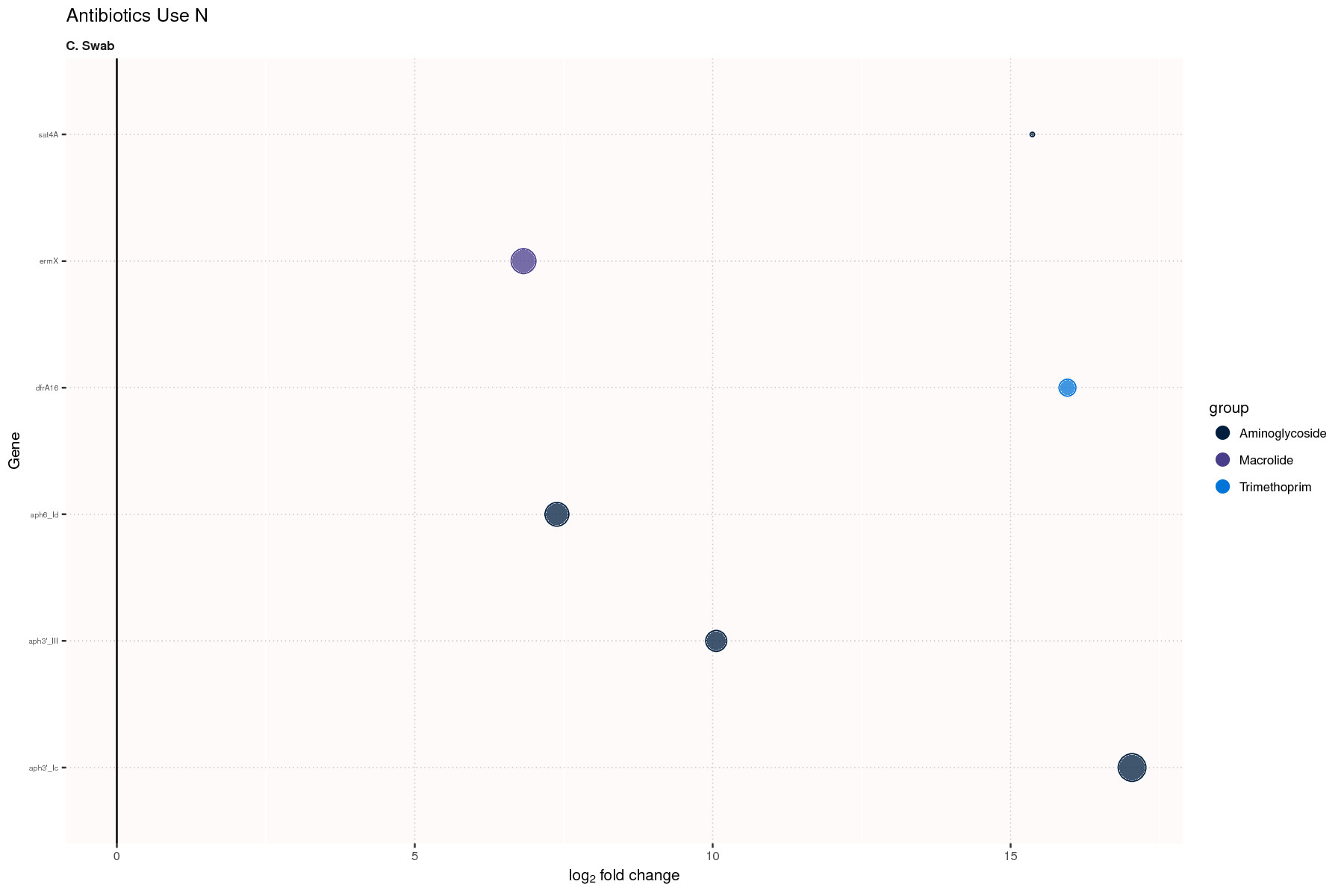

Supplement: FIGURE S3B — Antimicrobial resistance genes showing more than a two-fold log change in prevalence in samples collected from infants in the new NICU compared to the old NICU samples, in infants who did not receive antibiotics. [file Image_6.JPEG]

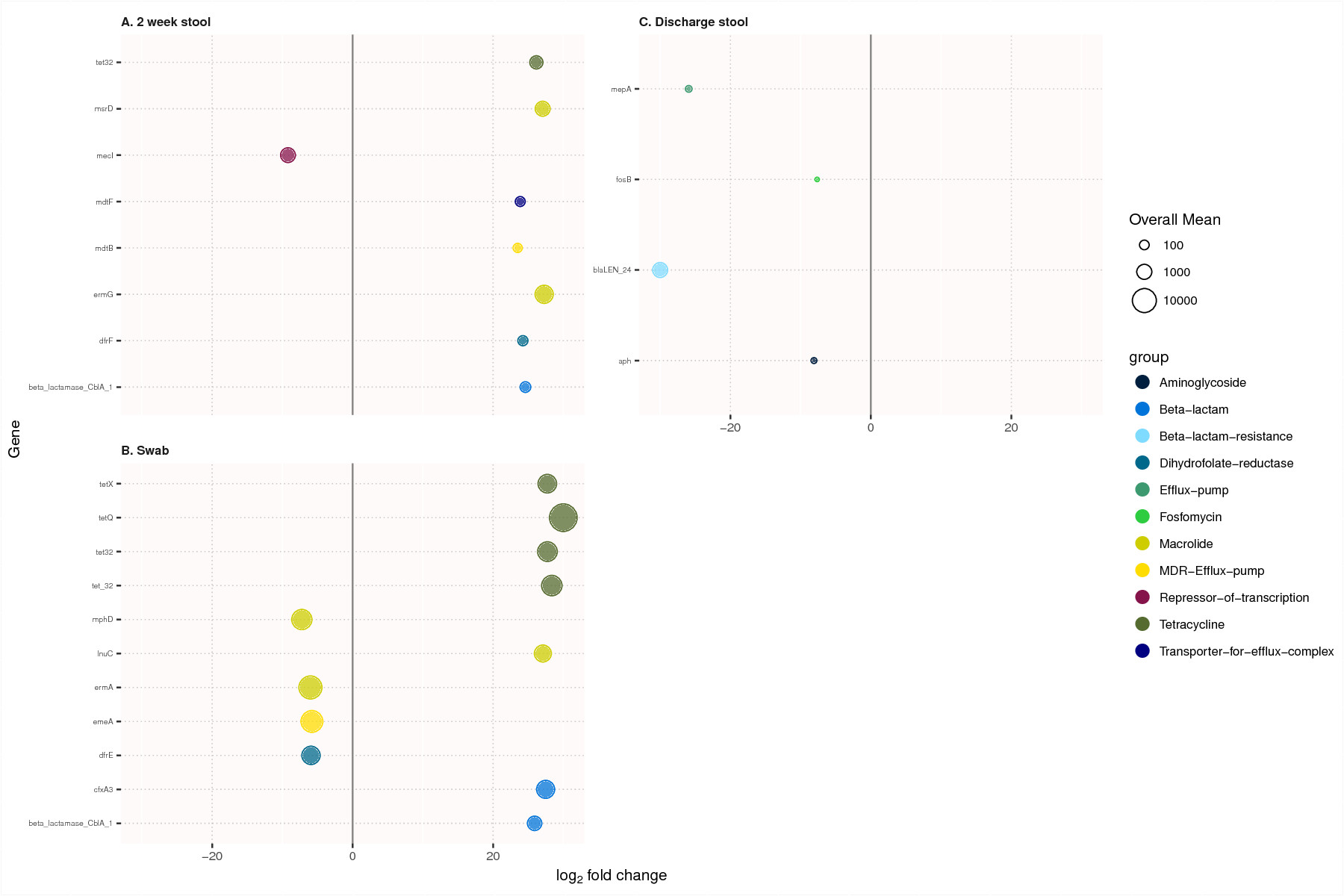

Supplement: FIGURE S4A — Antimicrobial resistance genes showing more than a two-fold log change in prevalence in samples collected from infants in the new NICU compared to the old NICU samples, in infants who primarily received breast milk. [file Image_7.JPEG]

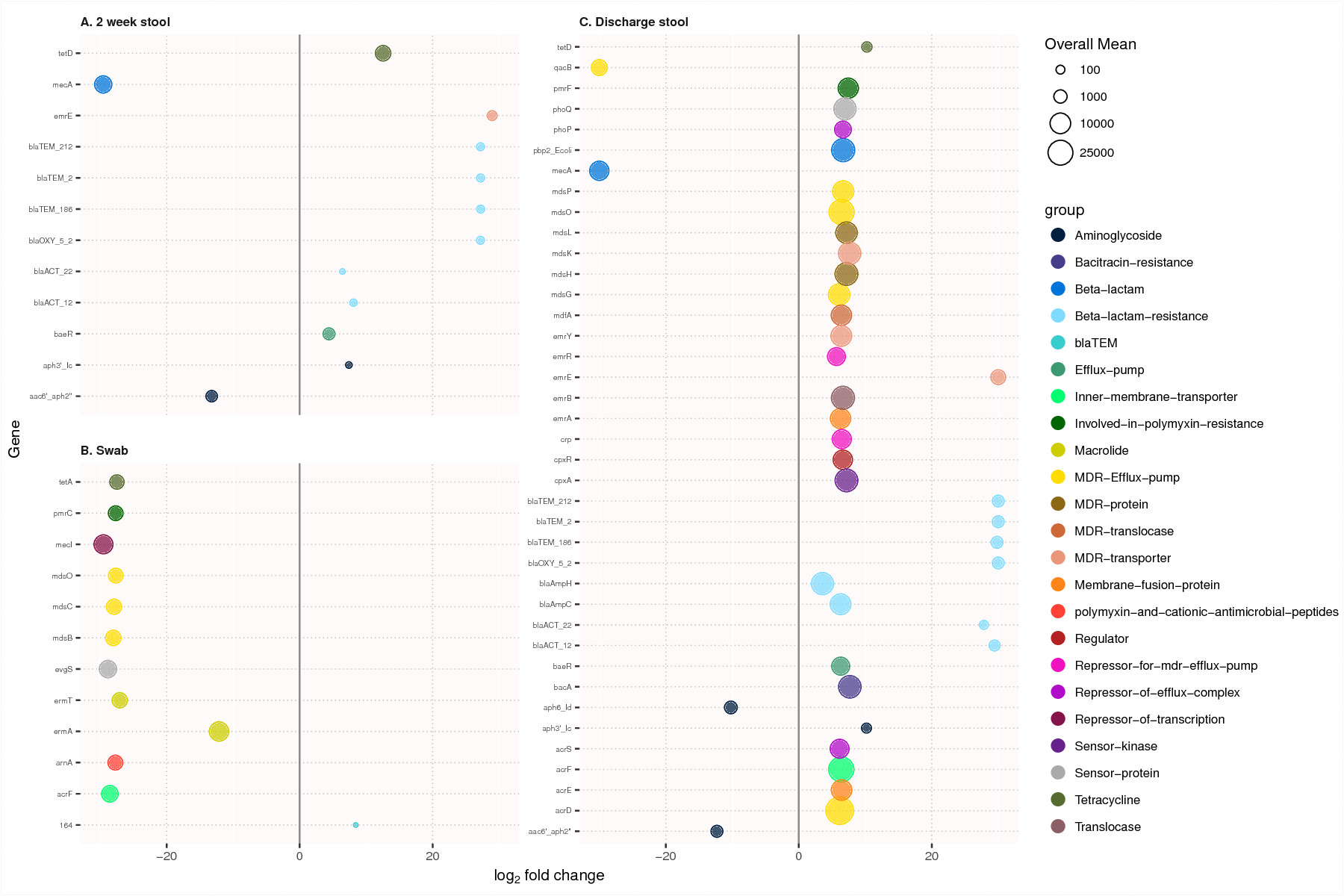

Supplement: FIGURE S4B — Antimicrobial resistance genes showing more than a two-fold log change in prevalence in samples collected from infants in the new NICU compared to the old NICU samples, in infants who did not primarily receive breast milk. [file Image_8.JPEG]

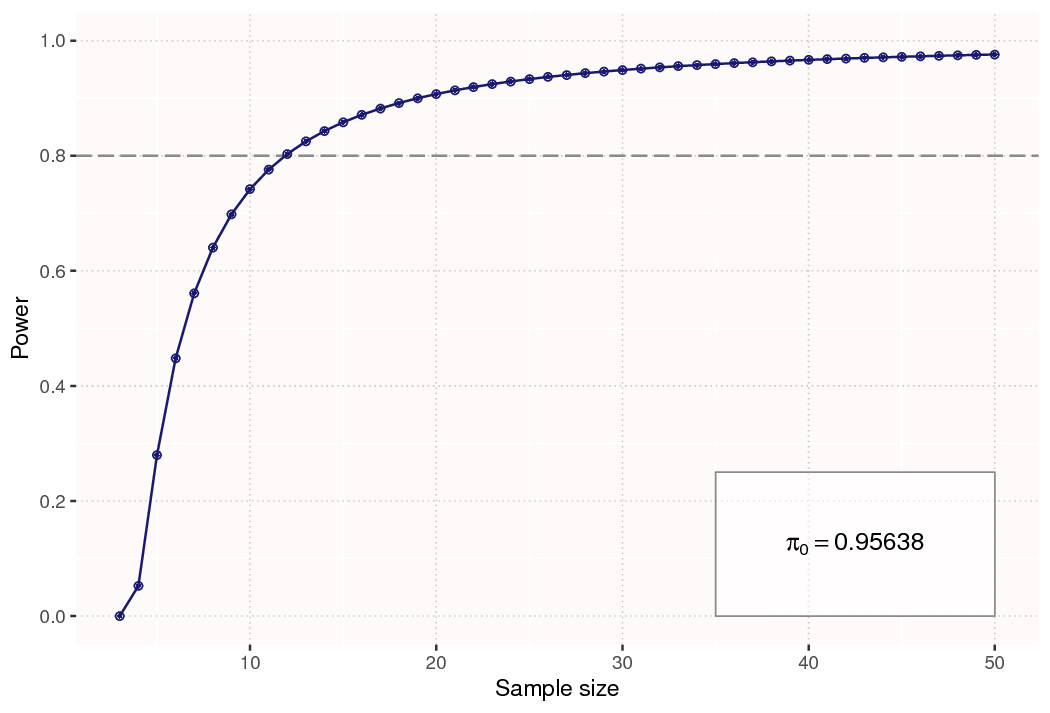

Supplement: FIGURE S5 — Average power versus sample size for differential gene abundance test using false discovery rate of 0.05. π0 is the estimated proportion of genes which are not differentially abundant. [file Image_9.JPEG]
